# Effectiveness of the 23-Valent Pneumococcal Polysaccharide Vaccine (PPV23) against Pneumococcal Disease in the Elderly: Systematic Review and Meta-Analysis

**DOI:** 10.1371/journal.pone.0169368

**Published:** 2017-01-06

**Authors:** Gerhard Falkenhorst, Cornelius Remschmidt, Thomas Harder, Eva Hummers-Pradier, Ole Wichmann, Christian Bogdan

**Affiliations:** 1 Department for Infectious Disease Epidemiology, Robert Koch Institute, Berlin, Germany; 2 Department of General Practice, University Medical Center Göttingen, Göttingen, Germany; 3 Mikrobiologisches Institut – Klinische Mikrobiologie, Immunologie und Hygiene, Friedrich Alexander Universität (FAU) Erlangen-Nürnberg and Universitätsklinikum Erlangen, Erlangen, Germany; Instituto Butantan, BRAZIL

## Abstract

**Background:**

Routine vaccination of elderly people against pneumococcal diseases is recommended in many countries. National guidelines differ, recommending either the 23-valent polysaccharide vaccine (PPV23), the 13-valent conjugate vaccine (PCV13) or both. Considering the ongoing debate on the effectiveness of PPV23, we performed a systematic literature review and meta-analysis of the vaccine efficacy/effectiveness (VE) of PPV23 against invasive pneumococcal disease (IPD) and pneumococcal pneumonia in adults aged ≥60 years living in industrialized countries.

**Methods:**

We searched for pertinent clinical trials and observational studies in databases MEDLINE, EMBASE, Cochrane Central Register of Controlled Trials, and Cochrane Database of Systematic Reviews. We assessed the risk of bias of individual studies using the Cochrane Risk of Bias tool for randomized controlled trials and the Newcastle-Ottawa Scale for observational studies. We rated the overall quality of the evidence by GRADE criteria. We performed meta-analyses of studies grouped by outcome and study design using random-effects models. We applied a sensitivity analysis excluding studies with high risk of bias.

**Results:**

We identified 17 eligible studies. Pooled VE against IPD (by any serotype) was 73% (95%CI: 10–92%) in four clinical trials, 45% (95%CI: 15–65%) in three cohort studies, and 59% (95%CI: 35–74%) in three case-control studies. After excluding studies with high risk of bias, pooled VE against pneumococcal pneumonia (by any serotype) was 64% (95%CI: 35–80%) in two clinical trials and 48% (95%CI: 25–63%) in two cohort studies. Higher VE estimates in trials (follow-up ~2.5 years) than in observational studies (follow-up ~5 years) may indicate waning protection. Unlike previous meta-analyses, we excluded two trials with high risk of bias regarding the outcome pneumococcal pneumonia, because diagnosis was based on serologic methods with insufficient specificity.

**Conclusions:**

Our meta-analysis revealed significant VE of PPV23 against both IPD and pneumococcal pneumonia *by any serotype* in the elderly, comparable to the efficacy of PCV13 against *vaccine-serotype* disease in a recent clinical trial in elderly people. Due to its broader serotype coverage and the decrease of PCV13 serotypes among adults resulting from routine infant immunization with PCV13, PPV23 continues to play an important role for protecting adults against IPD and pneumococcal pneumonia.

## Introduction

Community-acquired pneumonia (CAP) is a major cause of hospital admissions and death in the elderly, with *Streptococcus pneumoniae* (pneumococcus) being the most frequently detected pathogen causing an estimated 20–30% of CAP cases [1, 2]. Invasive pneumococcal disease (IPD), in the elderly mostly presenting as pneumonia with bacteremia, is the most severe form of pneumococcal infections. Case fatality can exceed 20% in elderly patients [3]. More than 90 serotypes of *S*. *pneumoniae* can be distinguished on the basis of the antigen structure of the capsular polysaccharide.

Two pneumococcal vaccines are currently licensed for adults: one containing polysaccharides from 23 pneumococcal serotypes (PPV23), the other containing protein-conjugated polysaccharides from 13 serotypes (PCV13). Most industrialized countries recommend universal pneumococcal vaccination for the elderly, but there is considerable debate about the best vaccination strategy [4]. The choice of vaccine is primarily determined by the efficacy (i.e. the protective effect assessed in randomized controlled trials (RCTs)) or effectiveness (i.e. the protective effect assessed in observational studies) of the two vaccines against pneumococcal pneumonia (PP) and IPD, as well as by the prevalence of the pneumococcal serotypes contained in the respective vaccine among cases. In addition, cost-effectiveness aspects may be considered.

The pivotal RCTs leading to the license of the first commercial pneumococcal vaccine, a 14-valent plain polysaccharide vaccine (PPV14), were conducted in the 1970s among gold miners in South Africa, a population group with a high incidence of PP [5, 6]. In 1983, PPV14 was replaced by PPV23. Since then, its efficacy/effectiveness in the elderly has been investigated in several RCTs and observational studies.

We performed a systematic review and meta-analyses of RCTs and observational studies investigating the efficacy of PPV23 against the specific outcomes PP and IPD in people aged ≥60 years living in industrialized countries.

Since we started our review, three other systematic reviews and meta-analyses of PPV23 efficacy/effectiveness have been published in the beginning of 2016 [7–10]. Prior to these publications, a Cochrane review from 2013 [11] presented the most up-to-date meta-analysis. Remarkably, these four reviews have come to divergent conclusions regarding clinical effectiveness of PPV23. We scrutinized these reviews and discovered that they have ignored a major methodological flaw in two large efficacy trials of PPV23, likely resulting in in an underestimation of the efficacy of PPV23 against PP. We believe that our work not only helps to resolve the discrepancies between previously published meta-analyses, but also highlights the importance of a meticulous appraisal of the risk of bias of published VE studies.

## Methods

We systematically assessed the evidence on the efficacy/effectiveness of PPV23 against clinical endpoints in the elderly, employing the following steps:

1. We reviewed all studies that were assessed in the most comprehensive systematic review published so far, including studies that ultimately did not meet the inclusion criteria for the meta-analysis [11].
2. We then updated the literature search of that review, and meta-analyzed all relevant studies, excluding studies with a high risk of bias in a sensitivity analysis.
3. Finally, we compared results of our meta-analysis with those of the other recently published reviews.

We followed the Preferred Reporting Items for Systematic Reviews and Meta-analysis (PRISMA) statement [12]. Our review protocol is available as appendix S1 Text.

### Eligibility criteria

According to our predefined PICOS criteria (*Population*, *Intervention*, *Comparator*, *Outcome*, *Study design*, see Table 1), eligible studies had to be an original report on the efficacy or effectiveness of PPV23 in individuals aged 60 years and older. The control group had to have received placebo or no vaccine. We considered publications in which the specific clinical outcomes IPD or PP (or both) were assessed. We included clinical trials and observational studies, using the term *vaccine efficacy* for data from clinical trials, *vaccine effectiveness* for data from observational studies, and the abbreviation VE for either one or both, depending on context. Observational studies were only included if they reported VE estimates that were adjusted at least for age and comorbidities. No restrictions were made regarding publication language, and publication status. We excluded immunogenicity studies, studies with older PPV formulations containing more antigen per serotype (e.g. PPV14 with 50μg compared to 25μg in PPV23), and studies conducted in developing countries.

**Table 1:** PICOS criteria for eligibility of studies.

**P**opulation	Persons 60 years and over, healthy or with age-typical underlying diseases living in industrialized countries and not belonging to indigenous minority populations
**I**ntervention	Vaccination with PPV23
**C**omparator	No vaccination or placebo
**O**utcomes	IPD and PP
**S**tudy design	RCTs Observational studies, if adjusted at least for age and comorbidities

### Updated literature search

We used the review from the Cochrane Collaboration [11] as starting point and conducted an update literature search for subsequently published studies in the databases MEDLINE, EMBASE, and Cochrane Central Register of Controlled Trials from 01.01.2011 to 02.07.2015 with an adapted search strategy (S1 Table). Two reviewers (GF and CR) independently screened titles, abstracts and full text articles. In addition, reference lists of all identified studies and reviews were reviewed for additional studies. In case of discordances regarding literature screening process, data extraction, and quality assessment a final decision was made by consensus or resolved by a third reviewer (TH). We updated the literature search on 15.07.2016 and did not find additional studies.

### Data extraction

From each eligible study, two independent reviewers (GF and CR) extracted the following information using standardized forms: authors, publication year, study design, country, study population, number of participants, duration of follow-up, person-years of follow-up, reported outcomes, reported effect measure (RR; adjusted HR or OR), and funding. The extraction forms were pilot-tested with the first identified study of each study type and the field "person-years of follow-up" was added. The corresponding author of one study was contacted to clarify discrepancies in published data.

### Assessment of risk of bias and quality of the body of evidence

We used the Cochrane Risk of Bias tool [13] to assess risk of bias in randomized controlled trials (RCTs) and the Newcastle-Ottawa Scale for observational studies [14]. For each study, risk of bias by outcome was independently assessed by two reviewers (GF and CR) and expressed as considered judgment as either “low”, “high” or “unclear”. We judged the overall quality of the body of evidence using the *Grading of Recommendations Assessment*, *Development*, *and Evaluation (GRADE) Working Group* [15] criteria. In GRADE, bodies of evidence from RCTs are a priori regarded as "high" quality evidence, whereas those from observational studies start as "low" quality evidence. Defined criteria are applied to up- or downgrade the quality of evidence which is finally expressed as “high”, “moderate”, “low” or “very low” [15].

### Statistical analysis

Extracted data were aggregated in tables. Risk ratios (RR), adjusted odds ratios (aOR) and corresponding 95% confidence intervals (95% CIs) of clinical endpoints in the PPV23-vaccinated group and the control group were either directly extracted from the publications or calculated using person-years of follow-up as denominator. Vaccine efficacy/effectiveness (VE) was calculated as (1–RR)x100 or (1-aOR)x100, respectively. If in observational studies data for various periods of time since vaccination were reported, we used the data for a period of 5 years.

Meta-analysis using a random-effects model was performed by study design if data on a given outcome were available from more than one study. We used the software Review Manager (RevMan, version 5.2, Cochrane Collaboration), which offers two options for the statistical analysis of random-effects models: the Mantel-Haenszel method and the inverse variance method. In our case, both methods produced identical results. Between-study variation was estimated by comparing each study’s result with a Mantel-Haenszel fixed-effect meta-analysis result. For comparisons with zero events in any cell, the software automatically adds 0.5 to all cells. I-squared was used to quantify the extent of heterogeneity.

In the primary analysis, all eligible studies were included. According to the recommendations of the Cochrane Collaboration [16], we conducted sensitivity analyses including only studies with a low risk of bias. Testing for publication bias was not done since study numbers for each outcome were too small. The results of the GRADE evidence rating were recorded in GRADE evidence profiles using the GRADEpro software [17].

### Funding

This systematic review was financed by the authors’ respective institutional budgets without extramural funding.

## Results

### Selection of studies

**Fig 1:**
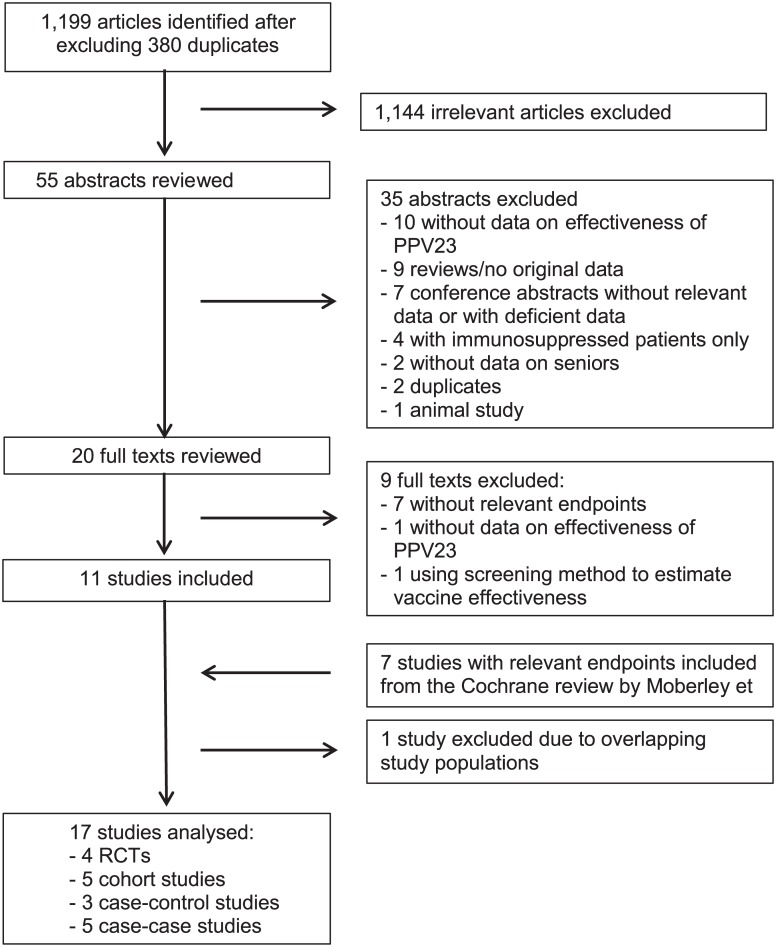
Flowchart of literature search.

Overall, 4 clinical trials (3 RCTs and one pseudo-randomized trial) and 13 observational studies were included (Fig 1). Of those, 7 studies derived from the review of the Cochrane Collaboration [11] and further 10 studies were identified with the updated literature search in electronic databases. Screening of the reference lists of included studies did not reveal any additional eligible studies.

### Characteristics of included studies

The three RCTs [18–20] were conducted between 1991 and 2009 in Sweden, Spain, and Japan and included 596 to 1006 participants (Table 2). The pseudo-randomized trial [21] was conducted in Finland and included almost 27,000 participants.

**Table 2:** Characteristics of studies included in the systematic review of PPV23 efficacy/effectiveness.

Publication	Study type	Country	Study population	Number of vaccinated/unvaccinated	Period of follow-up observation	Sponsor	Risk of bias	Inclusion for endpoints IPD/PP
Alfageme 2006 [18]	RCT	Spain	COPD patients; median age vaccine group 69, unvaccinated group 68, range 61–73 years	298/298	2.7 years	Spanish Pneumology Society, Andalusian Health Service	Low	Y/Y
Maruyama 2010 [19]	RCT	Japan	Nursing home residents; mean age vaccine group 84.7, placebo group 84.8, range 55–105 years	502/504	2.3 years	Japanese Ministry of Education, Culture, Sports, Science, and Technology	Low	Y/Y
Örtqvist 1998 [20]	RCT	Sweden	Former CAP patients; mean age vaccine group 69.4, placebo group 69.1, range 50–85 years	339/352	2.4 years	Pasteur-Mérieux MSD, Swedish Heart-Lung Foundation, Karolinska Institute	Low ^a^	Y/N^a^
Honkanen 1999 [21]	(RCT) ^b^	Finland	Resident population aged ≥65 years; mean age vaccine group 73.3, unvaccinated group 73.7 years	13,980/12,945	1.4 years	Academy of Finland, Pasteur-Mérieux	Unclear^a^	Y/N^a^
Hechter 2012 [25]	Cohort	USA	Participants of the longitudinal *California Men's Health Study*, aged ≥60 years	7,718/9,232 at study begin	Variable	Kaiser Permanente Southern California	High	Y/N^c^
Jackson 2003 [24]	Cohort	USA	Resident population, aged ≥65 years	42,977/84,203 (PY)	Variable (81% 5–8 years)	CDC (USA)	Low	Y/N^c^
Ochoa-Gondar 2014 [22]	Cohort	Spain	Resident population, aged ≥60 years	29,065/46,968 (PY)	up to 5 years	Spanish Health Ministry	Low	Y/Y
Tsai 2015 [26]	Cohort	Taiwan	Resident population, aged ≥75 years	229,181/229,181	1 year	Taiwan CDC	High	Y/N^c^
Vila-Corcoles 2006 [23]	Cohort	Spain	Resident population, aged ≥65 years	17,401/16,504 (PY)	Variable (87% 2–5 years)	Spanish Health Ministry	Low	Y/Y
				**Cases/Controls**				
Dominguez 2005 [27]	Case-control	Spain	VT IPD cases ≥65 y + matched controls	131/393	2–3 years	Directorate of Public Health, Catalonia	Low	Y/N^d^
Leventer-Roberts, 2015 [29]	Case-control	Israel	IPD cases ≥65 y + matched controls	212/848	up to 5 years	Pfizer	Low	Y/N^d^
Vila-Corcoles 2009 [28]	Case-control	Spain	IPD and PP cases ≥50 y (74% ≥65 y) + matched controls	IPD: 94/188 PP: 304/608	up to 7.5 years	Spanish Health Ministry	Low	Y/Y
Andrews 2012 [30]	Case-case	England & Wales	IPD cases ≥65 y	444/369 ^e^	up to 5 years	Health Protection Agency	Low	Y/N^d^
Gutiérrez 2014 [33]	Case-case	Spain	IPD cases ≥60 y	588/211 ^e^	up to 5 years	No information	Low	Y/N^d^
Rudnick 2013 [32]	Case-case	Canada	IPD cases ≥65 y	1138/240 ^e^	up to 5 years	Canadian Institutes for Health Research, CDC USA, Ontario Thoracic Society, Abbott Laboratories, Bayer Healthcare, GlaxoSmithKline, Pfizer	Low	Y/N^d^
Wright 2013 [31]	Case-case	England	IPD cases ≥65 y	374/73 ^e^	up to 9 years	Health Protection Agency, Sanofi Pasteur MSD	Low	Y/N^d^
Wiemken 2014 [35]	Case-case	USA, Europe	CAP cases ≥65 y	279/2409 ^f^	No information	No funding	High	N/Y

CAP = community-acquired pneumonia, IPD = invasive pneumococcal disease, PP = pneumococcal pneumonia, PY = person years follow-up,

VT IPD = vaccine type invasive pneumococcal disease, Y = yes, N = no

^a^ Endpoint PP excluded because the majority or all of the reported PP cases were diagnosed using insufficiently specific serologic tests for pneumolysin antibodies

^b^ Pseudo randomization according to birth year (even/uneven)

^c^ Endpoint PP not reported

^d^ Only IPD cases were included in the study.

^e^ IPD cases caused by vaccine serotypes / IPD cases caused by non-vaccine serotypes

^f^ CAP cases caused by pneumococci / CAP cases of other or unknown etiology

Five register-based cohort studies were conducted between 1998 and 2011 in Spain [22, 23], US [24, 25], and in Taiwan [24, 26] including 34,000 to 458,000 person-years of follow-up (Table 2).

Three case-control studies were conducted between 2001 and 2010 in Spain [27, 28], and Israel [29]. A variation of the case-control design, the so-called Broome method, was used in four studies from the UK [30, 31], Canada [32], and Spain [33], in total covering IPD surveillance data from 1995 to 2012. Across all case-control studies, 4320 episodes of IPD were included. With the Broome method [34], VE against IPD caused by vaccine serotypes (VT) is estimated by comparing vaccine uptake in patients with VT-IPD ("cases") and patients with non-VT-IPD ("controls"). As both groups consist of cases of disease, this study design is also known as "case-case study".

With a similar approach, one multi-country study [35] analyzed cases of PP, using pneumonia cases of other or unknown etiology as controls.

### Reported outcomes

VE against the outcome IPD was reported in all but one [35] study. The clinical trials, cohort studies, and classical case-control studies reported all-serotype IPD (i.e., IPD caused by any pneumococcal serotype). Two of the case-control studies also reported VT-IPD. The 4 studies using the Broome method reported estimates of VE against VT-IPD.

VE against all-serotype PP was assessed in 4 trials, in 2 cohort studies [22, 23], and in 2 case-control studies [28, 35]. In these studies, PP was diagnosed by a range of methods, including a urine-antigen test which does not allow differentiation of pneumococcal serotypes. Therefore, VE against PP caused by vaccine serotypes could not be calculated.

### Risk of bias assessment

#### Clinical trials

For the outcome IPD, we rated the risk of bias as low for all clinical trials. In the pseudo-randomized trial by Honkanen et al. [21] group allocation was based on participants' year of birth (odd vs. even), and participants were not blinded as to their vaccination status. Moreover, they were offered to switch groups, which only 4.5% of participants did, however. It appears very unlikely that these methodological shortcomings decisively altered the chance of being diagnosed with IPD during the follow-up period.

For the outcome PP, we judged the studies by Örtqvist et al. and Honkanen et al. [20] [21] to have a high risk of bias: In these studies diagnosis of PP was made on the basis of detection of serum antibodies against pneumolysin using poorly validated in-house ELISA methods [36, 37]. These assays were later shown to have poor specificity, thus biasing the observed VE in a vaccine trial towards no effect (see Discussion for details).

#### Observational studies

10 of 13 observational studies were judged to have low risk of bias [22–24, 27–29, 31–33]. The remaining three studies were judged to bear a high risk of bias for the following reasons: In the study by Hechter et al. [25] participants were men who were voluntarily participating in a broader longitudinal study on men’s health (high risk of selection bias); in the study by Wiemken et al. [35] vaccination status of participants was not sufficiently validated (high risk of differential misclassification bias). In the study by Tsai et al. [26], VE against all-cause mortality was implausibly high at 93%, suggesting an over-estimation of VE (also against other outcomes) due to healthy vaccinee bias [38].

### Vaccine efficacy/effectiveness

#### Outcome IPD

**Fig 2:**
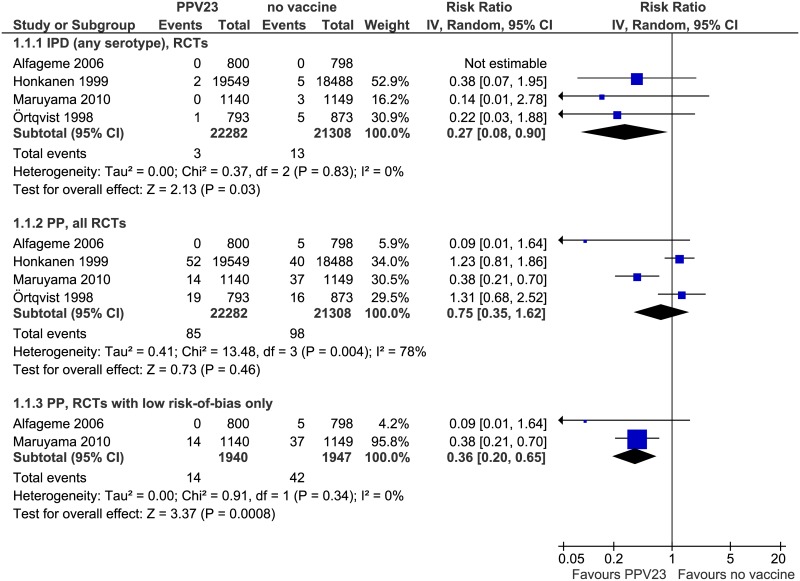
Forest plots of meta-analyses of randomized controlled trials, outcomes IPD and pneumococcal pneumonia. IPD = invasive pneumococcal disease PP = pneumococcal pneumonia RCT = randomized controlled trial.

Pooled analysis of all included clinical trials showed a VE of 73% (95% CI: 10–92%, I^2^ = 0%) against IPD with any serotype (Fig 2).

**Fig 3:**
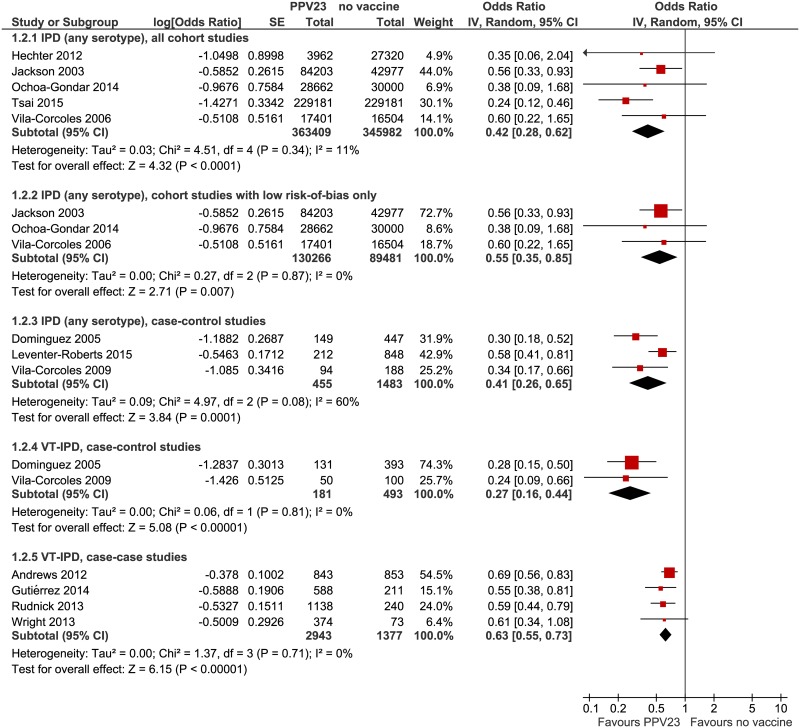
Forest plots of meta-analyses of observational studies, outcome IPD. IPD = invasive pneumococcal disease VT-IPD = vaccine-serotype IPD.

In cohort studies, pooled VE against IPD (any serotype) including all studies was 58% (95% CI: 38–72%, I^2^ = 11%), but decreased to 45% (95% CI: 15–65%, I^2^ = 0%) when studies with high risk of bias [25, 26] were excluded (Fig 3).

In case-control studies, pooled VE was 59% (95% CI: 35–74%, I^2^ = 60%) against IPD (any serotype). Heterogeneity is due to the lower VE observed in the study by Leventer-Roberts et al.. This study was conducted several years later than the other two studies, at a time when the proportion of vaccine-preventable serotypes among all IPD cases had probably already declined due to herd protection resulting from universal pneumococcal vaccination of infants. Effectiveness against *vaccine-type* IPD was only reported in the two older case-control studies with a pooled estimate of 73% (95% CI: 56–84%, I^2^ = 0%). Pooled analysis of case-case studies revealed VE of 37% (95% CI: 27–45%, I^2^ = 0%) against VT-IPD (Fig 3).

#### Outcome pneumococcal pneumonia (PP)

Pooled analysis of all included clinical trials showed a VE of 25% (95% CI: -62-65%) against PP (any serotype) with marked heterogeneity (I^2^ = 78%). After exclusion of studies with high risk of bias [20, 21], VE increased to 64% (95% CI: 35–80%) without heterogeneity (Fig 2).

**Fig 4:**
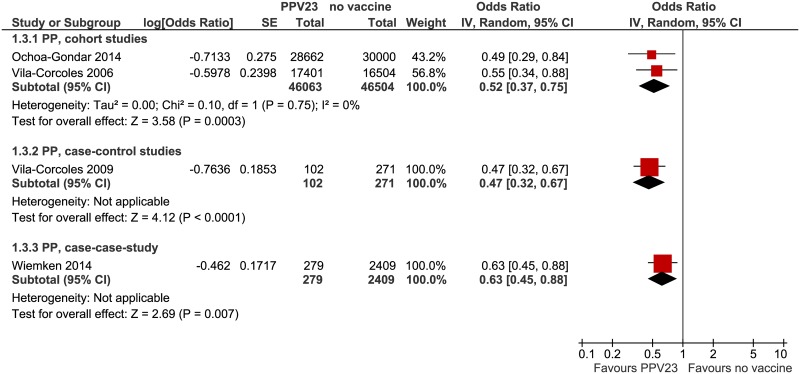
Forest plots of meta-analyses of observational studies, outcome pneumococcal pneumonia. PP = pneumococcal pneumonia.

Pooled analysis of the cohort studies showed a VE of 48% (95% CI: 25–63%, I^2^ = 0%) against PP. Of the remaining observational studies, only one case-control study [28] (VE 53%, 95% CI: 33–68%) and one case-case study with high risk-of-bias [35] (VE 37%, 95% CI: 12–55%) reported on that outcome (Fig 4).

### Quality of the evidence

The quality of the evidence for both outcomes (IPD, PP) was assessed as *moderate* (the second highest level in the GRADE system) on the basis of data from RCTs (incl. the pseudo-randomized trial [21]). Reasons for downgrading by one level were wide CIs (GRADE criterion *imprecision*) in the case of IPD. For the outcome PP, the quality was downgraded because evidence is mainly based on one trial done in very old and frail nursing home residents [19] in whom the VE may be different (probably lower) than in the general population aged ≥60 years (GRADE criterion *indirectness*) (see S2 Table).

### Comparison of this review with recently published meta-analyses

As shown in Table 3, previous systematic reviews of PPV23 efficacy/effectiveness have used different inclusion criteria for study selection and different outcomes. This partially explains why they reached divergent conclusions regarding clinical effectiveness of PPV23, in particular against pneumonia. Kraicer-Melamed et al. [8] used residence in nursing homes as an exclusion criterion, thereby excluding the Japanese RCT by Maruyama et al. [19]. Similarly, Schiffner-Rohe et al. [10] excluded that RCT in their stratified analysis. None of the review authors discusses the validity of serologic tests for the diagnosis of PP, as used in the trials by Örtqvist et al. [20] and Honkanen et al. [21].

**Table 3:** Overview of recent meta-analyses of PPV23 efficacy/effectiveness.

	**Inclusion criteria**			**Pooled vaccine efficacy/effectiveness (95% CI)**				
Author, year	Age group (years)	Study design	Included studies ^1^	IPD, any serotype	Pneumococcal pneumonia, any serotype	All-cause CAP	All-cause mortality	Declared conflicts of interest
Moberley, 2013 [11]	'adults'	RCTs	A M Ö	74% (55 to 86) ^2^	54% (16 to 75) ^2^	28% (7 to 44) ^2^	10% (-9 to 26) ^2^	None
		Obs. studies		52% (39 to 63) ^2^	NR	NR	NR	
Kraicer-Melamed, 2016 [8, 9]	50+ (excl. nursing home residents)	RCTs	H Ö	NR	range -28% to -20% ^3^	-10% (-36 to 12)	NR	1 of 3 authors received research funding from GSK and Pfizer for unrelated projects
		Cohort		50% (21 to 69)	range 5% to 45% ^3^	17% (-26 to 45)	NR	
		CaCo		54% (32 to 69)	48% (27 to 63) ^4^	7% (-10 to 21)	NR	
Diao, 2016 [7]	15+	RCTs	A M Ö	NR	46% (-65 to 82)	13% (2 to 24)	-4% (-24 to 13)	None
		Obs. studies	none					
Schiffner-Rohe, 2016 [10]	65+	RCTs	A H M Ö	NR	incl. M: 28% (-58 to 67) excl. M: -11% (-93 to 36)	-10% (-30 to 7)	NR	All authors employed by Pfizer (manufacturer of PCV13 vaccine) or by a Pfizer contractor
		Obs. studies	none					
Our meta-analysis	60+	RCTs	A H M Ö	73% (10 to 92)	incl. H+Ö: 25% (-62 to 65) excl. H+Ö: 64% (35 to 80)	NR	NR	None
		Cohort		45% (15 to 65) ^5^	48% (25 to 63)	NR	NR	
		CaCo		59% (35 to 74)	53% (33 to 68) ^4^	NR	NR	

CAP = community acquired pneumonia, CaCo = case-control study, excl. = excluding, incl. = including, IPD = invasive pneumococcal disease, NR = not reported, Obs. = observational, RCT = randomized controlled trial

^1^ A = Alfageme et al. (2006), M = Maruyama et al. (2010), Ö = Örtqvist et al. (1998), H = Honkanen et al. (1999). Additional RCTs were included in meta-analyses regarding outcomes other than IPD and pneumococcal pneumonia, and those including age groups younger than 60 years.

^**2**^ Including studies conducted with older PPV formulations containing a higher amount of antigen per serotype (e.g. PPV14)

^3^ no pooled estimate reported

^**4**^ only one study

^5^ excluding studies with high risk of bias

## Discussion

Our systematic literature review and meta-analysis revealed that PPV23 is effective against both IPD and PP (caused by any pneumococcal serotype) in the elderly. The point estimates of vaccine efficacy—derived from the meta-analysis of RCTs with low risk of bias—were 73% against IPD and 64% against PP (25% when including studies with high risk of bias). These estimates are supported by results from observational studies with low risk of bias. The pooled vaccine effectiveness against IPD (any serotype) in the first 5 years after vaccination was 45% in cohort and 59% in case-control studies, against PP it was 48% and 53%, respectively. These somewhat lower estimates may indicate waning of protection over the years, as the follow-up in the two RCTs of high quality lasted only 2.3 and 2.7 years, respectively, but on average 5 years in observational studies.

The question whether or not plain polysaccharide vaccines such as PPV23 can protect against PP is the subject of controversial discussions [4, 39, 40]. Historically, efficacy against PP has been clearly demonstrated in the 1970s in RCTs among workers in the gold mines in South Africa. In that population of young men, efficacy of 6- and 12-valent PPV against PP was 76% and 92%, respectively [6]. These results cannot be readily applied to the vaccination of an elderly population with the current 23-valent vaccine, but they provide a proof of principle. As further detailed below, efficacy/effectiveness of PPV23 against PP has been demonstrated in different settings and with different study designs, strongly suggesting that it is a real effect despite methodological limitations of individual studies.

### Comparison with previous systematic reviews

Regarding efficacy against IPD, our results are in accordance with previous meta-analyses addressing this outcome [8, 11]. Regarding the outcome PP, the pooled VE of our meta-analysis of clinical trials with a low risk of bias was similar to that reported by Moberley et al. [11]. Contrarily, the latest meta-analyses [7–10] found no statistically significant VE against PP. Their estimates were driven by the trials by Örtqvist et al. [20] and Honkanen et al. [21], see Table 3.

We judged the trials by Örtqvist et al. and Honkanen et al. to have a high risk of bias regarding VE against the outcome PP, because diagnosis of PP was made by detection of antibodies against pneumolysin, a cholesterol-dependent cytotoxin produced by almost all strains of *S*. *pneumoniae*. In both trials, pneumolysin antibodies in serum (Ply-serum) and in circulating immune complexes (Ply-IC) were measured at the National Public Health Institute in Finland, using poorly validated in-house ELISA methods [36, 37]. These assays have not been used for the diagnosis of PP in any published study by other groups, nor have they become part of clinical routine. The main problem is their lack of specificity, which biases the observed effect in a VE study towards the null [39, 41, 42]. In the original publication by Jalonen et al. [36], specificity of the Ply-serum assay is not reported. In their validation study of the Ply-IC assay, Leinonen et al. [37] observed that geometric mean antibody titers measured with the Ply-serum ELISA were higher in healthy controls than in pneumonia patients, raising serious doubt about the specificity of the Ply-serum ELISA. For the Ply-IC assay, they report a specificity of 83%. However, as their healthy comparison group was much younger than their pneumonia patients, the validity of that specificity estimate is dubious.

The authors (including Leinonen) of a later validation study of the Ply-IC ELISA concluded that sensitivity and specificity of the assay were "insufficient for the performance of analytical epidemiological investigations or vaccine efficacy studies" [41]. A validation study of the Ply-IC ELISA by an independent group came to a similar result [42]. Moreover, that group showed that detection of antibodies to pneumolysin does not allow to differentiate between infection and mere colonization. Specificity of pneumolysin serology for the diagnosis of pneumococcal infection is further compromised by the fact that in addition to *S*. *pneumoniae* pneumolysin is expressed by other alpha-hemolytic streptococci such as *S*. *viridans* [43].

Another important difference between our and previous reviews is the in- or exclusion of the RCT by Maruyama et al. [19]. Kraicer-Melamed et al. [8] and Schiffner-Rohe et al. [10] *ex*cluded this trial, arguing that the study population of nursing home residents was not representative of the general elderly population. However, the same authors *in*cluded the trial by Örtqvist et al. [20], which was carried out in patients who had recently been treated in hospital for pneumonia. The representativeness of these patients for the general elderly population is equally questionable.

### Limitations

Our meta-analysis of the efficacy against PP rests on only two RCTs with a low risk of bias and is dominated by the larger Japanese study by Maruyama et al. [19]. This study was undertaken in a population of very old, frail nursing home residents with an unusually high incidence of PP (32 per 1000 person-years in the placebo group). This study population is certainly not representative for the entire Japanese population aged 60 years and older. However, there is no biological reason to assume that the vaccine will be less effective in elderly people living outside nursing homes in Japan or other industrialized countries, as these vaccine recipients will be on average younger and have fewer comorbidities than nursing home residents. Furthermore, two register-based cohort studies among the resident elderly population in Tarragona, Spain, also showed a statistically significant VE against PP among persons vaccinated within the last 5 years [22, 23].

Another limitation for the interpretation of our data is the wide confidence intervals around the pooled VE estimates, leaving some uncertainty about the degree of protection. Also, the available data is insufficient to precisely determine the duration of protection afforded by PPV23.

### Choosing the right vaccine

Most industrialized countries recommend routine pneumococcal vaccination for the elderly. In the USA, the *Advisory Committee on Immunization Practices (ACIP)* recommends sequential vaccination with PCV13 followed by PPSV23 [44], whereas in the UK the *Joint Committee on Vaccination and Immunisation (JCVI)* recommends PPV23 only [45]. In Europe, some countries recommend sequential vaccination, others the use of PPV23 or PCV13 only, yet others (e. g. France, The Netherlands) do not advocate routine vaccination of healthy elderly at all (http://vaccine-schedule.ecdc.europa.eu).

PCV13 was originally developed for young children whose immature immune system lacking splenic marginal zone B cells and circulating IgM^+^ memory B cells does not respond well to plain polysaccharide antigens during the first 2 years of life [46]. In 2011, PCV13 was also licensed for use in adults on the basis of immunogenicity studies. Its efficacy against clinical endpoints in immunocompetent elderly was subsequently examined in a single randomized placebo-controlled trial in the Netherlands (CAPITA trial [47]). In the *modified intention-to-treat analysis* of that trial, efficacy of PCV13 against IPD and PP caused *by vaccine serotypes* was 76% (95% CI: 47–90%) and 38% (95% CI: 14–55%), respectively. These estimates are similar to our pooled VE estimates of PPV23 efficacy against IPD and PP *by any serotype*. VE of PCV13 against IPD and PP *by any serotype* was lower, reaching only 49% (95% CI: 21–67%) and 22% (95% CI: 2–39%), respectively [47]. However, for two reasons the CAPITA trial might overestimate the VE of PCV13 in the general elderly population: (i) Persons with immunocompromising conditions and those residing in nursing homes were not eligible; therefore, the CAPITA study population was in better health and possibly mounted a better immune response to the vaccine than the overall elderly population. (ii) The trial was conducted in 2008–2012, before the introduction of PCV13 for infant vaccination in the Netherlands. Hence, VE against IPD and PP *by any serotype* was observed at a time, when the proportion of PCV13 serotypes among cases of all ages was still high. In countries using PCV13 for infant immunization, a marked reduction of IPD cases by PCV13 serotypes has been seen in all age groups due to herd protection, reducing the potential benefit of PCV13 for the elderly [48–51]. For example in Germany, the proportion of PCV13 serotypes among IPD cases in ≥60 year old patients dropped from ~60% in the 2010/2011 season to ~30% in the 2015/2016 season, when still ~70% of cases were caused by serotypes included in PPV23 (www.rki.de/pneumoweb).

Data on the serotype distribution among cases of non-bacteremic PP is scarce, because often no isolate is available for serotyping. Serotype-specific assays for the detection of pneumococcal antigens in urine have only recently been developed and are so far limited to the 13 serotypes contained in PCV13 [52, 53]. A study of non-bacteremic PP cases in adults (median age 71 years) in Nottingham/England has shown a 30% reduction of the proportion of PCV13 serotypes within 3 years of the switch from PCV7 to PCV13 in the infant immunization program [54]. In a similar study in Germany, 79% of bacteremic PP and 62% of non-bacteremic PP cases were caused by PCV13 serotypes in the period 2007–2011, i. e. 3 years before and 2 years after the switch from PCV7 to PCV13 [55]. If anything, it appears that PCV13 serotypes are less prevalent among non-bacteremic PP than among bacteremic PP cases. Data covering a more recent time period is highly desirable to judge the further impact of infant immunization with PCV13 on serotype distribution in adult non-bacteremic PP.

### Conclusion

Our systematic review and meta-analysis indicates that PPV23 is effective against IPD and pneumococcal pneumonia in the elderly. In view of its broader serotype coverage compared to PCV13, PPV23 should be recommended for routine vaccination of the elderly. Sequential vaccination with PCV13 followed by PPV23 may be justified in countries where a large proportion of pneumococcal disease in the elderly is caused by PCV13 serotypes.

Regarding future research, an RCT directly comparing the efficacy of different vaccination strategies (PPSV23 only, PCV13 only, and sequential vaccination) on clinical endpoints is highly desirable. In addition, more data on the duration of protection by either vaccine as well as data on the optimal age for vaccinating elderly people would be useful.

## Supporting Information

S1 Table Search strategy. (DOCX)

S2 Table GRADE profile. (DOCX)

S3 Table PRISMA checklist. (DOCX)

S1 Text Protocol for the systematic review. (DOCX)
