# Supplementary material for: Effectiveness of the 23-Valent Pneumococcal Polysaccharide Vaccine (PPV23) against Pneumococcal Disease in the Elderly: Systematic Review and Meta-Analysis
Source: PLoS One. 2017 Jan 6;12(1):e0169368. doi: 10.1371/journal.pone.0169368 (PMC5218810; doi:10.1371/journal.pone.0169368)
Supplement: S1 Table — (DOCX) [file pone.0169368.s001.docx]

**S1 Search strategy**

Search strategy for the systematic review entitled

“Effectiveness of the 23-valent pneumococcal polysaccharide vaccine (PPV23) against pneumococcal disease in the elderly: systematic review and meta-analysis”

#1 “pneumococc*”

#2 “streptococcus pneumoniae”

#3 #1 OR #2

#4 “vaccine*”

#5 “immuni*”

#6 “polysaccharide vaccin*”

#7 “ppv”

#8 #4 OR #5 OR #6 OR#7

#9 “effectiveness”

#10 “efficacy”

#11 #9 OR #10

#12 #3 AND #8 AND #11

Restrictions: species: human

Databases searched: MEDLINE, EMBASE and Cochrane Central Register of Controlled Trials
from 1 January 2011 to 15 July 2016.
