# Supplementary material for: Effectiveness of the 23-Valent Pneumococcal Polysaccharide Vaccine (PPV23) against Pneumococcal Disease in the Elderly: Systematic Review and Meta-Analysis
Source: PLoS One. 2017 Jan 6;12(1):e0169368. doi: 10.1371/journal.pone.0169368 (PMC5218810; doi:10.1371/journal.pone.0169368)
Supplement: S2 Table — (DOCX) [file pone.0169368.s002.docx]

**S2 GRADE profile**

**GRADE profile : Efficacy of PPV23 in elderly people**

**Question:** Should PPV23 vs. no vaccination be used for prevention of IPD and pneumococcal pneumonia in people aged ≥60 years?

**Date:** 15 July 2016

**Settings:** industrialized countries

| **Quality assessment** | | | | | | | **Study size cases/person-years** | | **Effect** | | **Quality** |
| --- | --- | --- | --- | --- | --- | --- | --- | --- | --- | --- | --- |
|  |  |  |  |  |  |  |  |  |  |  |  |
| **No of studies** | **Design** | **Risk of bias** | **Inconsistency** | **Indirectness** | **Imprecision** | **Other considerations** | **PPSV23** | **no vaccination** | **Relative (95% CI)** | **Absolute** |  |
| **IPD all serotypes** (follow-up 1.4 to 2.7 years) | | | | | | | | | | | |
| 4 | **RCT** | no serious risk of bias^1^ | no serious inconsistency | no serious indirectness | serious^2^ | none | 3/22282  (0.01%) | 13/21308  (0.06%) | RR 0.27  (0.08 to 0.9) | 445 fewer per 1,000,000 (from 61 fewer to 561 fewer) | +++ MODERATE |
| **Pneumococcal pneumonia** (follow-up 2.3 to 2.7 years) | | | | | | | | | | | |
| 2 | **RCT** | no serious risk of bias | no serious inconsistency | serious^3^ | no serious imprecision | none | 14/1940  (0.72%) | 42/1947  (2.2%) | RR 0.36  (0.2 to 0.65) | 13806 fewer per 1,000,000 (from 7550 fewer to 17257 fewer) | +++ MODERATE |

^1^ Pseudo randomization according to birth year (even/uneven) and lack of allocation concealment in one trial, but unlikely to decisively alter the probability of the outcome.

^2^ Wide confidence interval (CI), absolute effect differs by factor ~9 at the limits of the 95% CI.

^3^ Indirectness in the sense that the study populations of both trials were not representative of the residential population aged ≥60 years. As the combined study populations were on average older and had more comorbidities, vaccine efficacy in the residential population aged ≥60 years may be higher.
