# Supplementary material for: Effectiveness of the 23-Valent Pneumococcal Polysaccharide Vaccine (PPV23) against Pneumococcal Disease in the Elderly: Systematic Review and Meta-Analysis
Source: PLoS One. 2017 Jan 6;12(1):e0169368. doi: 10.1371/journal.pone.0169368 (PMC5218810; doi:10.1371/journal.pone.0169368)
Supplement: S1 Text — (DOCX) [file pone.0169368.s004.docx]

**Protocol for systematic review:
Effectiveness of the 23-valent pneumococcal polysaccharide vaccine (PPV23) against pneumococcal disease in the elderly**

Falkenhorst G. et al.

1. **Review question:** What is the efficacy/effectiveness of PPV23 against pneumococcal disease in people aged ≥60 years?
2. **Data sources:**
   1. List of included and excluded studies of the Cochrane Review ' Vaccines for preventing pneumococcal infection in adults.' (Moberley et al. 2013)
   2. Electronic data bases: Medline, Embase, Cochrane Central Register of Controlled Trials, Cochrane Database of Systematic Reviews (from 1 January 2011 to date of search)
   3. Reference lists of all identified studies
   4. Restrictions: none regarding language or publication type
3. **Search strategy:** The following search strategy will be applied, using the full-text search option via DIMDI:

#1 pneumococc* OR streptococcus pneumoniae

#2 vaccin* OR immuni*

#3 effectiveness OR efficacy

#4 #1 AND #2 AND #3

#5 #4 AND py≥2011

#6 #5 AND species=human

1. **Population/participants:** Persons 60 years and over, healthy or with age-typical underlying disease, living in industrialized countries and not belonging to indigenous minority populations
2. **Intervention:** Vaccination with PPV23
3. **Comparator:** Placebo or no vaccination
4. **Outcomes:** Invasive pneumococcal disease (IPD), pneumococcal pneumonia (PP).
5. **Study designs:**
   1. RCTs
   2. Observational studies, if adjusted at least for age and comorbidities
6. **Data extraction:** Two independent reviewers (GF, CR) will screen studies by title and abstract for eligibility. Potential disagreements will be solved by discussion or by involving a third reviewer (TH). Identified studies will be retrieved in full text. The following data will be extracted, using standardized data extraction forms: Authors, publication year, study design, country, study population, number of participants (grouped by intervention or outcome status, respectively), duration of follow-up, person-years of follow-up, reported outcomes, reported effect measure (RR; adjusted HR or OR), and funding. Data extraction forms will be pilot tested with the first identified study of each study type and adapted as needed.
7. **Risk of bias assessment:** For randomized controlled trials, risk of bias will be assessed using the Cochrane Risk of Bias Tool. For observational studies, the Newcastle-Ottawa Scale will be applied.
8. **Data synthesis:** Basic characteristics of all included studies will be compiled in a table. For studies of identical study type (RCT or cohort study or case-control study or case-case study), pooled effect estimates will be calculated by meta-analysis using random-effects models. Meta-analyses will be performed including all eligible studies, using ReviewManager software. In addition, as a sensitivity analysis, meta-analyses will be repeated after exclusion of studies with a high risk of bias. Heterogeneity will be assessed using I² statistics. Funnel plots will be created to test for publication bias, if 10 or more studies of identical type are available.
9. **Quality assessment:** The overall quality of the evidence will be assessed by GRADE criteria and presented as GRADE profiles.
